# 

**Published:** 1880-01-20

**Authors:** 


### TABLE OF CONTENTS.

ORIGINAL AND SELECTED ARTICLES.

The Action and Uses of Digitalis, by A. G. Hobbs, M.D., of Indiana, 1. Some of the Cheap Preparations of the Bark—Cinchonia Alkaloid—Dextro-Quinine, by T. B. Greenley, of Kentucky, 5. Loss of Hair, by John V. Shoemaker, A.M., M.D., 6. Some Cases of Epistaxis and other Hemorrhages, by Randolph Winslow, M.D., 10. The Cure of Consumption, 13. Antiseptic Transfusion of Human Blood, by William Macewen, M.D., 15.

ABSTRACTS AND GLEANINGS.

Lactic Acid in Catarrh of the Bladder, 17. Meniere's Disease, 18. Obstetrics, 20. Calomel in Zymotic Diseases of Infancy, 21. Effects of Local Irritation on Pain; On Croup, 22. Ergotine Suppositories in Fibroid Tumors of the Uterus, 23. Popular Nostrums; Surgical Treatment of Goitre, 25. Unusual effect of a Hypodermic Injection of Morphia, 26. Intra-Tympanic Injections, 27. Traumatic Tetanus; German Treatment of Placenta Prævia, 28. Tooth Brushes; Metric System; A Daring Operation, 29. Gall Stones; Hypodermic Injections of Ether in Sciatica; Propylamine in Acute Rheumatism; Nearsightedness and the Color of the Eyes, 30.

SCIENTIFIC ITEMS.

Dangers of the Telephone; Night Temperature at Different Altitudes, 31. Philosophical Toy; New Use for Electricity; Annual Deaths of the World; Electrical Polyscope, 32.

PRACTICAL NOTES AND FORMULÆ.

Conversion of Calomel into Corrosive Sublimate; Cold Cream without Oil; Prostatic Enlargement; Tasteless Saline Purgatives, 33. Rheumatism; Whooping Cough Mixture; Ovarian Dysmenorrhœa; Quinia and Iron tonic, 34. Syphilis; Discutient Ointment; Asthma, 35. Vomiting during Pregnancy; Linimentum Potassii Iodidi; Cough Mixture; Flatulent Dyspepsia, 36.

EDITORIAL AND MISCELLANEOUS.

Editorial Notices, 37. Book Notices, 38. Receipted; Special Notices, 40.

